# Strain-Specific Behavior of *Mycobacterium tuberculosis *in Interruption of Autophagy Pathway in Human Alveolar Type II Epithelial A549 Cells

**DOI:** 10.52547/ibj.3586

**Published:** 2022-08-24

**Authors:** Nasim Ebrahimifard, Shima Hadifar, Mansour Kargarpour Kamakoli, Ava Behrouzi, Sharareh Khanipour, Abolfazl Fateh, Seyed Davar Siadat, Farzam Vaziri

**Affiliations:** 1 Department of Mycobacteriology and Pulmonary Research, Pasteur Institute of Iran, Tehran, Iran;; 2 Microbiology Research Center (MRC), Pasteur Institute of Iran, Tehran, Iran;; 3 Department of Microbiology, Faculty of Advanced Science and Technology Tehran Medical Science, Islamic Azad University, Tehran, Iran

**Keywords:** A549 cells, Autophagy, MicroRNA, Mycobacterium tuberculosis

## Abstract

**Background:**

Autophagy induction has been shown to differ in magnitude depending on the mycobacterial species. However, few studies have investigated the specific autophagic capacity of different *Mtb* strains in ATs. This study aimed to elucidate the host autophagic response to different *Mtb* strains in ATs responsible for TB in the capital of Iran, Tehran.

**Methods:**

A549 cells were infected with three different *Mtb* clinical isolates (Beijing, NEW1, and CAS1/Delhi) and the reference strain H37Rv. Following RNA extraction, the expression of eight ATG genes, four mycobacterial genes, and three miRNAs was evaluated using quantitative RT-PCR.

**Results:**

The results revealed that all four strains influenced the autophagy pathway in various ways at different magnitudes. The Beijing and H37Rv strains could inhibit autophagosome formation, whereas the CAS and NEW1 strains induced autophagosome formation. The expression of genes involved in the fusion of autophagosomes to lysosomes (LAMP1) indicated that all the studied strains impaired the autophagolysosomal fusion; this result is not unexpected as *Mtb* can block the autophagolysomal fusion. In addition, the Beijing and H37RV strains prevented the formation of autophagic vacuoles, besides mycobacterial targeting of lysosomes and protease activity.

**Conclusion:**

This preliminary study improved our understanding of how *Mtb* manages to overcome the host immune system, such as autophagy, and evaluated the genes used by specific strains during this process. Further studies with a large number of *Mtb* strains, encompassing the other main *Mtb* lineages, are inevitable.

## INTRODUCTION

*Mycobacterium tuberculosis,* as the causative agent of TB infection, is transmitted by inhaled aerosols^[1]^. After inhalation, *Mtb* strains, which can pass through the upper airways, will be delivered to the alveoli. The ATs, as non-professional phagocytic cells, are one of the first host cells that encounter *Mtb* in the alveoli^[2]^. These cells play an important role in the activation and uptake of immune cells, such as macrophages and neutrophils, into the site of infection by secreting cytokines and chemokines, which are necessary to establish infection^[3,4]^. ATs are lined with the alveolar lung mucosa, composed of an ALF that secretes complement and SPs, essential for the host immunity against *Mtb*^[5]^. The complex immunological events between AT and *Mtb* can be used for therapeutic strategies, especially in HDT^[6]^.

Autophagy is a complex and multistage process that includes initiation, elongation/closure, and maturation^[7,8]^. In each step of the autophagy pathway, various *atg* are involved^[9]^. The autophagic elimination of mycobacteria occurs when an LC3-IIB-positive autophagosome fuses with a lysosome to form an autolysosome, and the molecules and/or organisms sequestered in this vesicle are subjected to enzymatic degradation, subsequent recycling, and clearance of mycobacterial species. Inhibition of autophagy differs substantially among varied species^[10,11]^; therefore, focusing on autophagy may be helpful in introducing and developing cellular targets^[12]^.

Since some mycobacterial strains are more virulent than others, it is reasonable to hypothesize that pathways, such as autophagic response in ATs, may be different^[13,14]^. The Beijing strains have a more remarkable ability to escape autophagy, probably due to their higher capacity to inhibit autophagolysosome biogenesis upon autophagy induction compared to the reference strain, H37Rv. The ability of Beijing strains to evade the host autophagy may have important implications for TB treatment, especially in regions with common Beijing genotypes^[15]^. Recent findings have shown that the Beijing strain changes the position of the lysosome. The importance of lysosome repositioning as a new autophagy evasion strategy is related to the fact that it provides a new target for drug discovery against the disease^[16]^. Besides, emerging evidence suggests that miRNAs play key roles in regulating autophagy^[17]^; this effect is often established through the autophagic machinery. 

The possible application of miRNAs in the diagnosis and development of therapeutics for bacterial infections has been described^[18,19]^. In this study, we aimed to investigate the host autophagic response in an alveolar epithelial cell line (A549) in terms of key autophagy-related miRNAs and mycobacterial genes in response to infections caused by different *Mtb* strains (Beijing, NEW1, CAS1-Delhi, and H37Rv).

## MATERIALS AND METHODS

**Bacterial strains**

Three *Mtb* strains, including NEW1, CAS1-Delhi, and Beijing genotypes, were used in this study. These genotypes have previously been identified as the dominant genotypes of *Mtb* in Tehran, Iran, based on 24-locus MIRU-VNTR and spoligotyping^[20,21]^. The H37Rv strain was used in all assays as the reference strain. Briefly, all *Mtb* strains were grown in the Middlebrook 7H9 culture medium (Sigma Aldrich, St. Louis, MO, USA), supplemented with 10% albumin dextrose catalase (Becton Dickinson, Oxford, UK) at an OD_600_ of 0.6-0.9 at 37 °C.

**Cell culture treatment**

The A549 human lung epithelial cells (ATCC CCL‐185) were maintained in DMEM, supplemented with penicillin, streptomycin, 1% nonessential amino acids, and 10% fetal bovine serum (Gibco, Paisley, UK) in six-well plates (Sorfa, Zhejiang, China) in a 5% CO_2_ atmosphere at 37 °C. The confluent (60-70%) A549 cell line was infected by the clinical and reference *Mtb* strains at MOI ≈ 50:1. After two hours, the infected cells were rinsed twice with 1× phosphate-buﬀered saline to remove the extracellular bacteria and incubated in 5% CO_2_ at 37 °C for 72 hours. The viability of uninfected and infected cell lines was evaluated by the Trypan blue exclusion test, based on the manufacturer’s instructions (Sigma Aldrich, Germany).

**Intracellular growth assay**

The CFU/mL of the infected cultures were investigated at 72 hours post infection, as described previously^[22]^.

**RNA extraction, cDNA synthesis, and quantitative real-time PCR assay**

Total RNA was extracted from the infected, mock, and bacterial cells by TRIzol Reagent (Life Technologies Corp., Carlsbad, USA), according to the manufacturer’s instructions. The cDNA was synthesized using PrimeScript RT Reagent Kit (Takara, Japan), in accordance with the instructions provided by the manufacturer. qPCR was also performed using 2× SYBR Premix Ex Taq II (Tli RNase H Plus Takara, Japan) with a LightCycler® 96 qPCR instrument (Roche Applied Science, Penzberg, Germany). A reverse transcription reaction was performed using a FIREScript RT cDNA Synthesis Kit (Solis BioDyne, Estonia) to detect the miRNA levels, as per the manufacturer’s protocol. The reverse transcription reaction mixture consisted of total RNA (0.1 ng-5 µg), stem‐loop RT primer, U6 RT primer, 20 mM dNTP Mix, 10× reverse transcription reaction buffer, and FIREScript RT. The qPCR assay was performed with the LightCycler® 96 qPCR instrument (Roche Applied Science). The PCR volume included 1 µl of RT product, 1× SYBR Green real-time PCR Master Mix, and 0.5 µM of each forward and reverse primer. The PCR cycles were as follows: 95 °C for three minutes, followed by 40 cycles of 95 °C for 10 seconds, and 62 °C for 30 seconds. Each assay was performed in biological triplicates. The list of primer sequences used in this study is presented in Supplementary Table 1. 

**ELISA assay**

At 72 hour post infection, the supernatants of the treated and mock cells were collected, filtered through 0.22-μm syringe filters (Sorfa) and stored at -80 °C until further use. The level of IL-1β was measured by an ELISA kit (Biosciences, CA, USA) according to the instructions recommended by the manufacturer. The assay was performed in duplicate.

**Statistical analysis**

The relative gene expression was analyzed based on the 2^-ΔΔCt^ method. The *gapdh *gene for the cell line, the *secAl* gene for bacteria, and the *U6* gene for miRNA expression analysis were used as the reference genes. GraphPad Prism 8.0 (GraphPad Software Inc., CA, USA) was used for calculating changes in the gene expression and ELISA data. A *p* value less than 0.05 was considered statistically significant. The fold-change cut-off points for the comparison of down/upregulation between the control and infected groups were considered as <-2 and >2, respectively.

## RESULTS

**Intracellular growth assay and cell viability**

**Fig. 1:**
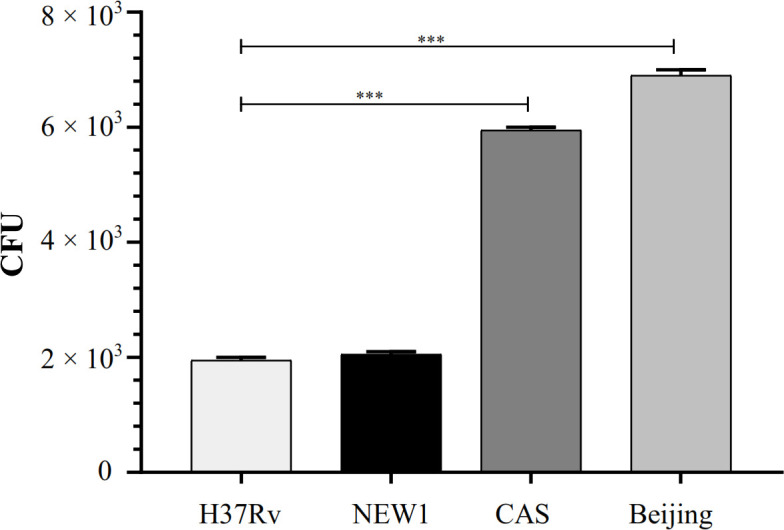
Intracellular growth of the studied *Mtb* genotypes in the A549 cell line at 72 h post infection. (^***^p < 0.001).

The results of CFU count in four studied genotypes at 72 hour post infection showed that the Beijing genotype had a greater intracellular load than other genotypes (Fig. 1). The effects of infection on cell viability showed that cell viability was >95%.

**Inducing different expression levels of autophagy-related genes** **in response to different *Mtb* genotypes**

**Fig. 2:**
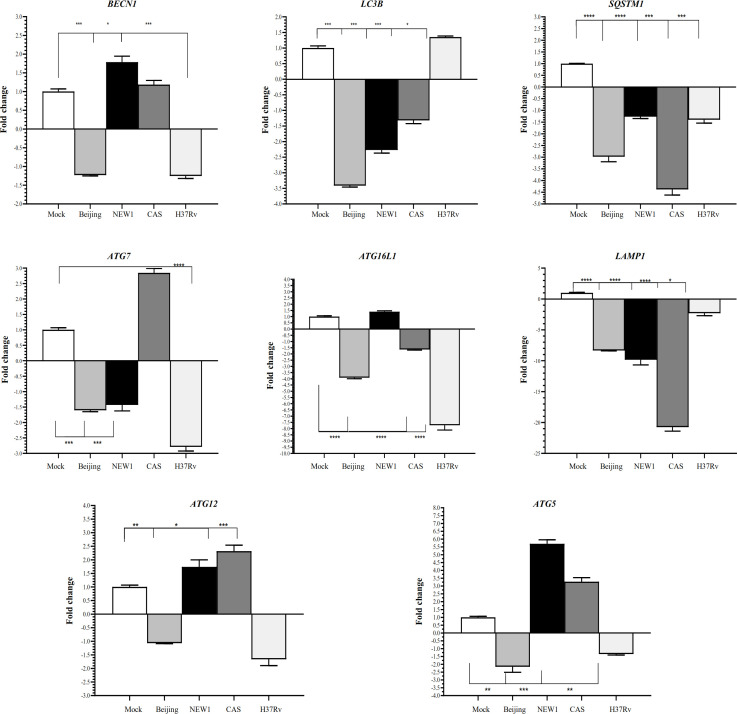
Effects of different *Mtb* genotypes on the mRNA expression of genes involved in autophagy in A549 cell line. Data are normalized using *GAPDH* as a control gene. ^∗^*p* < 0.05, ^∗∗^*p* < 0.01, and ^***^p < 0.001 are considered statistically significant compared to the mock cell.

To investigate the effect of different *Mtb* genotypes on the autophagy pathway in the A549 cell line, the expression of several genes, including *LC3B*,* ATG12*,* ATG5*,* ATG 16L1*,* ATG7*,* P62/SQSTM1* (sequestosome-1), *BECN1*, and *LAMP1*, was evaluated. As shown in Figure 2, the expression of all eight genes, except *BECN1*,* ATG12*, and *ATG7*, decreased in response to the Beijing (L2) genotype as compared to the mock cell (*p* < 0.05). In response to the CAS (L3) genotype, *P62/SQSTM1 *and *LAMP1* were downregulated, while *ATG12,* *ATG5*, and *ATG7 *upregulated (*p* < 0.05). In the infected cells by NEW1 (L4), the expression of *LAMP1* and *LC3B* was downregulated (*p* < 0.05), but *ATG5* upregulated. In response to infection with H37Rv (L4), as a reference strain, the expression of *ATG 16L1*, *ATG7*, and *LAMP1* genes decreased (*p* < 0.05), while no changes were observed in the expression of other genes. The results revealed that all the studied clinical *Mtb* strains differentially regulated the induction or inhibition of autophagy pathways. 

**Different expression levels of mycobacterial genes in the regulation of autophagy**

**Fig. 3:**
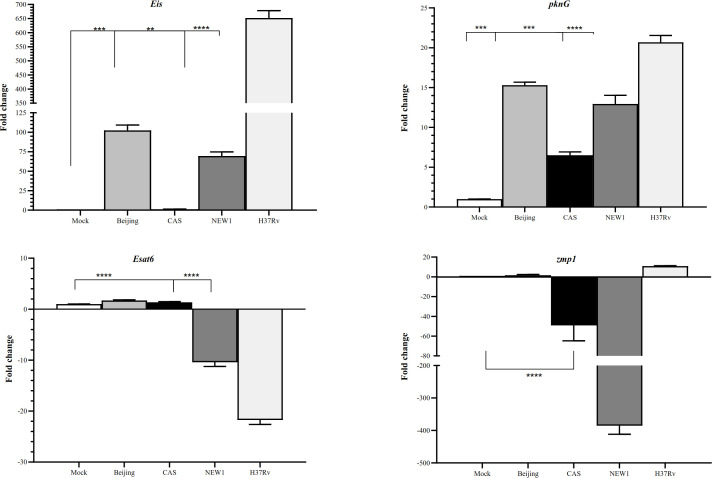
Expression of *eis*, *pknG*, *esat6*, and *zmp1* genes in the studied *Mtb* genotypes. Data are normalized using *secA *as the control gene. ^∗^*p* < 0.05, ^∗∗^*p* < 0.01, and ^***^p < 0.001 are considered statistically significant compared to the mock cell.

The present study evaluated the expression of four *Mtb* effectors (*eis*, *esat6*, *pknG*, and *zmp1*), which modulate the host autophagy pathways for survival and proliferation. The expression of *pknG* was upregulated in all studied *Mtb* genotypes compared to the *Mtb* strains grown in the Middlebrook 7H9 broth medium (Fig. 3). The expression of *eis* gene was upregulated in Beijing, NEW1, and H37Rv genotypes, while no change was observed in the CAS genotype. In NEW1 and H37Rv strains, the *ESAT6* expression decreased, and no change was found in Beijing and CAS. The expression of *zmp1* gene decreased in NEW1 and CAS genotypes, while it was upregulated in the H37Rv genotype.

**Different expression levels of miRNA genes related to autophagy in response to different** ***Mtb*** **genotypes**

**Fig. 4:**
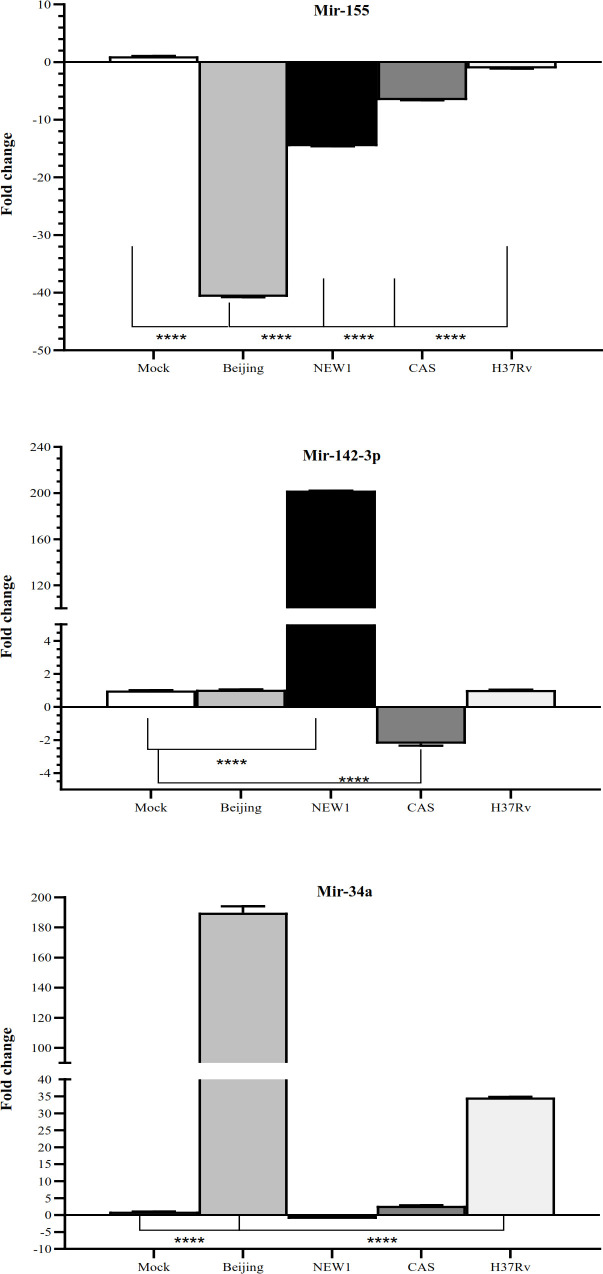
Expression of miR-155, miR-142-3p, and miR-34a in the infected A549 cells by the studied *Mtb* genotypes; Data are normalized using *U6 *gene as a control gene (^∗^*p* < 0.05, ^∗∗^*p* < 0.01, and ^***^p < 0.001 are considered statistically significant compared to the mock cell, respectively).

The expressions of miR-142-3p, miR-34a, and miR-155 were evaluated in response to infection induced by all of the studied genotypes. The expression of miR-155 decreased in response to Beijing, NEW1, and CAS, as compared to the mock cells (Fig. 4). The miR-142-3P was upregulated in the infected cells by NEW1, while it was downregulated in response to CAS. The expression of miR-34a was upregulated in response to all the studied genotypes, except for NEW1.

**Production of IL-1β in response to different** ***Mtb*** **genotypes**

The IL-1β secretion was mainly negative or very poor in response to infection by NEW1, CAS, and Beijing genotypes and mock cells. The level of IL-1β secretion in response to infection by H37Rv was 122.54 pg/mL.

## DISCUSSION

The fact that virulent mycobacteria have developed specific mechanisms to prevent autophagy highlights the importance of autophagy in response to *Mtb* infection^[23]^. The study of *Mtb *host interactions, considering the genetic background of strains, can provide in-depth insights into the mechanisms involved in the establishing infection and introducing targets, which can be useful for the treatment/prevention of TB infection^[13,14,24]^. Overall, the understanding of the mechanisms of autophagy during *Mtb* infection can be helpful for this purpose^[25]^. The analysis of differential gene expression following A549 infection with different *Mtb* strains indicated that several genes were up- or downregulated more than twofold. These autophagy-associated genes, which are up- or downregulated in response to infection with *Mtb*, would be possible candidate TB susceptibility genes. Previous studies have shown that *atg* genes control the autophagosome formation through Atg12-Atg5-ATG16L1 and LC3 complexes^[26]^. In this regard, Guo *et al.*^[25]^ knocked down Atg5 in A549 cell line to further evaluate the role of autophagy in *Mtb* infection. The knockdown of this protein led to a significant reduction in the LC3B protein expression in mock cells and confirmed the role of ATG5 in autophagosome maturation. Similarly, it has been shown that mice deficient in the central autophagy pathway components, such as ATG5, have a higher bacterial burden and increased inflammation, which appears in a cell-autonomous manner^[25]^. In the present study, the expression of *ATG16L1*,* ATG5*, and *LC3B* genes, as the main markers of autophagy, reduced in response to the Beijing genotype (*p* < 0.05). The Atg12-Atg5-Atg16 complex specified the site of LC3-PE production; this complex is involved in formation of an autophagosome membrane and led to phagophore expansion^[27]^. In infections by the NEW1 genotype, despite increased expression of *ATG5*, the expression of *LC3B* decreased (*p* < 0.05). Also, the CAS1 genotype induced the upregulation of *ATG5*,* ATG7*, and *ATG12* genes (*p* < 0.05). As *ATG5 *plays a pivotal role in the autophagosomal formation, the increased expression of *ATG5* in the NEW1- and CAS-infected cells (*p* < 0.05) revealed that the Beijing strain prevented autophagosomal membrane formation more efficiently than the NEW1 and CAS strains. The NEW1 and CAS strains may not be effective in preventing autophagosomal membrane formation once autophagy is induced. This characteristic provides an advantage for the development of Beijing genotype infections as compared to other studied genotypes. Besides, the expression of *ATG5* and *ATG16L1* genes is influenced by miR-142-3p. The miR-142-3p, by the inhibition of *ATG16L1* and downregulation of *ATG5*, causes a reduction in autophagosome formation and, therefore, interferes with the autophagy pathway and bacterial clearance^[28]^. Similarly, in our study, the expression of miR-142-3p decreased in the CAS1 strain, while the expression of *ATG5* increased (*p* < 0.05). In contrast, in the NEW1 strain, the *Atg5* expression increased (*p* < 0.05). This controversial finding may be justified by the downregulation of miRNA-155 in our study, which had inhibitory effects on the expression of *ATG5*. The study of Chen *et al.*^[29]^ also demonstrated that the inhibition of miR-155 increased the expression of *ATG5*; therefore, miR-155 modulated autophagy by decreasing the expression of *ATG5*. In our study, an increase in *ATG5* expression in the NEW1 strain might have occurred following a decrease in miR-155. Eskelinen *et al.*^[30]^ suggested that LAMP proteins play a pivotal role in the lysosomal integrity and maintain the lysosomal membrane against hydrolytic enzymes within the lysosome. In this respect, Huynh and colleagues^[31]^ have shown that LAMP1 and LAMP2 are needed for the autophagy fusion stage. In the present study, since all of the strains induced a decrease in the *LAMP1* expression (*p* < 0.05), it may be hypothesized that *Mtb* strains can hinder autophagosomal fusion with lysosomes by downregulating the *LAMP1* expression. 

The expression profile among the studied strains indicates a significant downregulation of autophagy-related genes in A549 cells infected with the Beijing strain, as compared to CAS and NEW1 strains, suggesting the more virulent *Mtb* strain more significantly affected the autophagy pathway compared to the less virulent strain.

Briefly, the Beijing strain is more armed to prevent the autophagy pathway as compared to the CAS and NEW1 strains. It can be assumed that this strain enables the formation of autophagic vacuoles and successfully inhibits the autophagolysosome fusion, enabling the pathogen's survival. 

The increased expression of *eis* gene as a bacterial gene, which prevents autophagy^[32]^, can be another proof for our hypothesis about the inhibition of autophagy in response to *Mtb* strains. It has been reported that *eis *has a significant role in infection outcomes and also the regulation of the host autophagic response by preventing the autophagic vacuole formation^[32, 33]^. This effect may be due to the e-amino acetyltransferase property of *eis*, with impacts on the JNK pathway. According to previous studies, since JNK is an important regulator of Beclin 1 and is also involved in regulating autophagy with Atg7 activity, it can be speculated that *eis* is responsible for inhibiting autophagy^[32,34]^. In the present study, the expression of *Atg7* gene also decreased (*p* < 0.05) following an increase in *eis *gene expression in the H37Rv genotype, which can confirm the mentioned finding. The increased expression of *pknG* gene is another factor that may reduce autophagy in our studied strains. A previous study suggests that PknG enhances mycobacterial virulence and proliferation within the host by mitigating the autophagy-mediated clearance through the phosphorylation of P62/ SQSTM1^[35]^. Changes in the expression of these two genes (*P62/SQSTM1 *and *pknG*) in the Beijing and CAS strains can be effective in reducing autophagy. The *P62/SQSTM1 *expression is not only affected by pknG but also is one of the miR-155 targets, which can also be altered by this miRNA. Chen *et al.*^[29]^ have demonstrated that when a miR-155 inhibitor is used, the expression of LC3 increases, and the expression of P62/SQSTM1 decreases. Similarly, in our study, in both Beijing and CAS strains, the expression of miR-155 decreased.

Similar to the results reported by Zhang and associates^[36]^, our findings revealed that the inhibition of autophagy is related to mycobacterial ESAT-6/CFP-10 effectors by affecting the reduction of *ATG8*,* ATG5*,* ATG7*, and *ATG12* expression. In the present study, autophagy is inhibited by decreasing the expression of *atg8 (LC3)* and *atg5* in Beijing strain. Chandra *et al.*^[37]^ implied that the virulence strains of *Mtb* prevented the maturation of autophagosomes with virulence factors, ESAT-6 and PhoP. This stage of autophagy requires a Rab7 marker. Another potential regulator of the retrieval step is the tumor suppressor, miR-34a, which has recently been identified as an inhibitor of the autophagic flux and a direct regulator of ATG9A and ATG4B in mammalian cells^[38,39]^. In our study, this miRNA increased in response to all the studied genotypes, except for NEW1. This increase can be another strategy that the *Mtb* genotypes utilized for disrupting the autophagy pathway. 

A limitation of this study is that we were unable to affirm the gene expression results via Western blotting or protein immunoblotting, which is a reliable and superior test to detect the relative protein expression. This technique was not performed in this study because antibodies were not available due to unforeseen problems with the import permission. In future studies, Western blotting is required to confirm gene expression results. 

To conclude, the present findings improved our understanding of how different *Mtb* genotypes (dominant strains in Tehran) manipulate the autophagy pathway. Further studies with other *Mtb* genotypes, host cells, and genes-related autophagy pathways can help to understand this pathway deeply and evaluate *Mtb* strain-specific behavior in host-pathogen interaction.  There are only few studies exploring the miRNA effects on the autophagic flux. Therefore, for an inclusive understanding of the regulator function of miRNAs in autophagy, further investigations are needed.

## DECLARATIONS

### Acknowledgments

We thank all the staffs of Mycobacteriology and Pulmonary Research Department, Pasteur Institute of Iran for their assistance in this project.

### Ethical statement

Not applicable.

### Data availability

Data supporting this article are included within the article.

### Author contributions

FV supervised the project. FV, SDS, and AF designed the project. FV and NE wrote the manuscript. MKK, SK, and SH performed laboratory work. SH and AV performed the statistical analysis. All authors have read and approved the final manuscript.

### Conflict of interest

The authors declare that they have no conflicting interests.

### Funding/support

This work was supported by a Ph.D. grant from Pasteur Institute of Iran and a grant (project no. 98000097) from Iran National Science Foundation (INSF).
